# Diagnosis and genetic analysis of Gaucher disease in a pediatric case: a case report

**DOI:** 10.3389/fped.2025.1628525

**Published:** 2025-07-28

**Authors:** Mengting Ma, Nan Wu, Jie Feng, Xu Sang, Feixiang Duan, Congcong Li, Qiang Zhang

**Affiliations:** ^1^ Department of Laboratory Medicine, The First Affiliated Hospital of Bengbu Medical University, Bengbu, China; ^2^ Molecular Diagnosis Center, The First Affiliated Hospital of Bengbu Medical University, Bengbu, China; ^3^ Department of Neurosurgery, The First Affiliated Hospital of Bengbu Medical University, Bengbu, China; ^4^ Department of Pediatrics, The First Affiliated Hospital of Bengbu Medical University, Bengbu, China; ^5^ Department of Radiology, The First Affiliated Hospital of Bengbu Medical University, Bengbu, China

**Keywords:** Gaucher disease, β-glucocerebrosidase, Mendelian inheritance, genetic testing, rare disease

## Abstract

A 2-year-old patient was admitted to our hospital with hepatosplenomegaly as the prominent clinical feature. Peripheral blood analysis during hospitalization revealed trilineage cytopenia. Bone marrow cytology examination demonstrated abundant suspected Gaucher cells. Full-spine MRI exhibited widening of the distal femoral metaphysis with an “Erlenmeyer flask deformity.” Subsequent enzymatic and genetic evaluations for Gaucher disease (GD) confirmed reduced β-glucocerebrosidase (GBA) activity, significantly elevated glucosylsphingosine (Lyso-Gb1) levels, and a homozygous missense mutation in the *GBA* gene c. 1448T>C(p.Leu483Pro). Genetic testing of the parents revealed both were heterozygous carriers of the same mutationc. 1448T>C(p.Leu483Pro), confirming the diagnosis of GD in the child with an autosomal recessive inheritance pattern. GD typically presents in childhood with hepatosplenomegaly, anemia, and thrombocytopenia. Given its rarity and nonspecific clinical manifestations, bone marrow cytology and imaging studies may provide diagnostic clues, but definitive diagnosis requires confirmation through β-glucocerebrosidase activity assays and genetic testing. Enzyme replacement therapy (ERT) is currently the primary treatment modality. The child is receiving regular intravenous infusions of imiglucerase at our hospital.

## 1 Introduction

The history of Gaucher disease (GD) dates back to 1882 when French dermatologist Philippe Gaucher first identified abnormal cells in a patient with splenomegaly. He later named these cells “Gaucher cells” and the disease “Gaucher disease” after himself (1, 2). In recent years, advancements in molecular genetics have confirmed that GD is caused by mutations in the *GBA* gene located on chromosome 1q21, leading to a deficiency in β-glucocerebrosidase activity (3). This deficiency results in the accumulation of its substrate, glucocerebroside, within the lysosomes of macrophages in the liver, spleen, bones, lungs, and even the brain, forming the characteristic Gaucher cells. Clinically, GD manifests as a multisystem disorder with varying degrees of organ involvement. GD is a rare disease, with a global incidence ranging from 0.39 to 5.8 per 100,000, varying by country. It is particularly prevalent among Ashkenazi Jews, North African Arabs, and certain Asian populations (Such as India and Southeast Asia) (4, 5). In China, the highest incidence rates are reported in Hebei, Shandong, and Henan provinces, with approximately 1,000 cases documented to date (6). This study presents a clinical and familial genetic analysis of a pediatric GD case with prominent hepatosplenomegaly, aiming to enhance clinicians' awareness of this rare condition and elucidate its genotypic-phenotypic correlations.

## 2 Case description

A girl child, 2 years old, was admitted to the hospital due to hepatosplenomegaly and fever lasting for 2 days without an obvious cause. During the course of the illness, the child exhibited mild lethargy, normal dietary intake, adequate sleep, and no significant changes in weight. Urination and defecation were normal. The parents reported no consanguineous marriage, no family history of genetic disorders, and both parents were in good health (though they had not undergone genetic testing for related diseases). The family also mentioned that the child had a habit of nail-biting. Upon admission, physical examination revealed: body temperature 39.3℃, heart rate 120 beats per minute, respiratory rate 25 breaths per minute. She was conscious, with symmetrical bilateral breath sounds that were slightly coarse. Her abdomen was flat and soft without tenderness. Her liver could be palpated 2 cm below the costal margin, and the spleen 4 cm below the costal margin. No abdominal masses or tenderness were detected, and no deformity of the spine or limbs. Neurological Examination: Extraocular movements are full, with intact horizontal saccade initiation. Nasolabial folds are symmetrical bilaterally. Knee jerks are graded ++ (normal). Muscle strength and tone are normal in all four limbs. Brudzinski's sign is negative. Kernig's sign is negative. Bilateral Babinski signs are negative.

### 2.1 Laboratory investigations

After admission, the patient underwent a series of laboratory tests, including complete blood count, biochemical profile, and viral nucleic acid detection. The laboratory findings revealed leukopenia, anemia, and thrombocytopenia. Elevated levels of alkaline phosphatase and lactate dehydrogenase were observed, while other liver function markers were within normal limits. The results of viral nucleic acid detection were all negative. The ESR and CRP levels were slightly elevated, indicating possible inflammation (Table 1).

**Table 1:** Laboratory investigations.

Investigation	Unit	Normal range	Measured value
White blood cell count	10^9^/L	4.4–11.9	3
Red blood cell count	10^12^/L	4–5.5	3.96
Hemoglobin	g/L	112–149	100
Platelet count	10^9^/L	188–472	48
Alanine Aminotransferase (ALT)	U/L	7–30	20
Aspartate Aminotransferase (AST)	U/L	14–44	66
Alkaline Phosphatase (ALP)	U/L	143–406	1,160
Gamma-Glutamyl Transferase (GGT)	U/L	5–15	21
Total Bilirubin	umol/L	2–22	9.8
Total Protein	g/L	61–79	72.9
Albumin	g/L	39–54	41.7
Potassium Ion	mmol/L	3.9–5.4	3.4
Sodium Ion	mmol/L	135–145	136
Creatine Kinase (CK)	U/L	30–325	87
Creatine Kinase Isoenzyme (CK-MB)	U/L	9.5–36.5	3
Lactate Dehydrogenase (LDH)	U/L	120–246	302
C-Reactive protein (CRP)	mg/L	0–10	10.04
Erythrocyte sedimentation rate (ESR)	mm/1h	0–20	31
EBV-DNA	Copies/m	<4.0E + 02	<400
RSV-RNA		Negative	Negative
Adv-DNA		Negative	Negative
Mp-DNA		Negative	Negative
FluA-RNA		Negative	Negative
FluB-RNA		Negative	Negative
HRV-RNA		Negative	Negative

### 2.2 Abdominal ultrasound examination

The abdominal ultrasound revealed the following findings:

• Liver: The maximum oblique diameter of the right lobe of the liver was 94 mm, with a regular contour and smooth capsule. The parenchymal echotexture was slightly heterogeneous, but the granularity appeared normal. The vascular architecture was clearly visualized, and the portal vein diameter measured 10 mm.
• Spleen: The spleen exhibited a thickness of 53 mm and a length of 136 mm. The parenchymal echotexture was uniformly distributed, and the splenic vein diameter measured 6 mm. No other significant abnormalities were observed.

### 2.3 Bone marrow aspiration cytology examination

The bone marrow aspiration cytology revealed the following findings:

• Cellularity: The nucleated cells in the entire smear exhibited markedly hyperplastic proliferation.
• Granulocytic Series: Accounted for 39% of the cells, with a notable scarcity of mature-stage cells, indicating maturation arrest.
• Erythroid Series: Comprised 15% of the cells, displaying essentially normal morphology and structure.
• Lymphoid and Megakaryocytic Series: Both lineages showed active proliferation. Platelets were sparsely distributed as single entities, suggesting a reduction in number.

Throughout the smear, a variable number of suspected Gaucher cells were observed. These cells were characterized by:

Size and Shape: Large, round, or oval-shaped cells.

**Figure 1:**
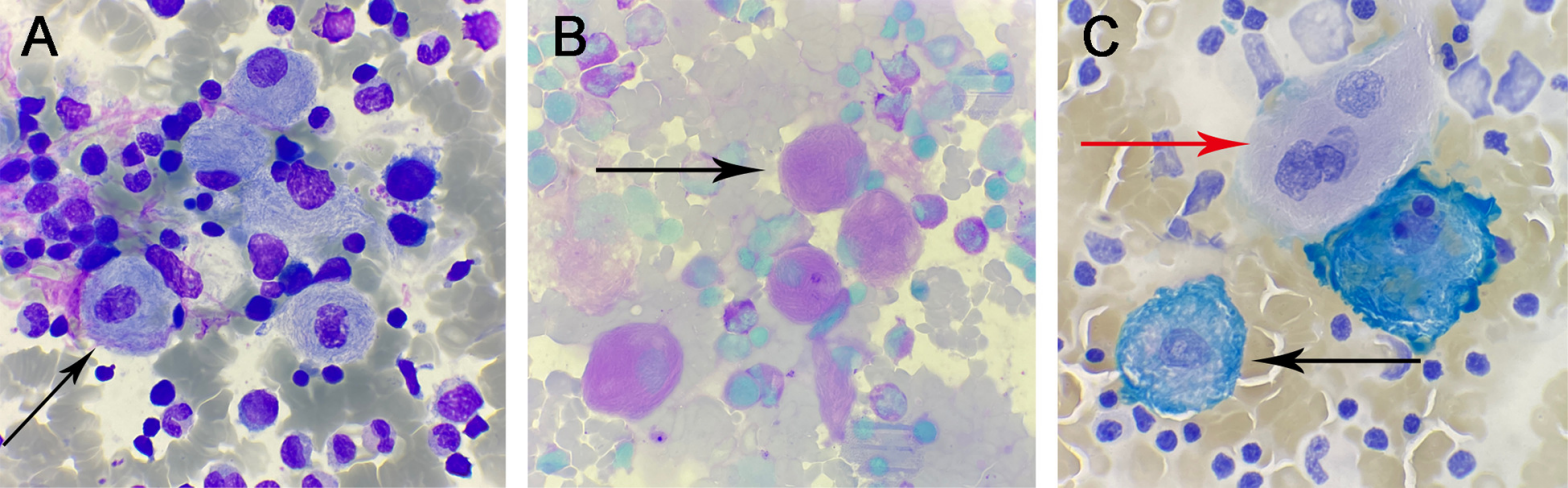
**(A)** Wright-Giemsa stain, ×1,000: Gaucher cells. **(B)** Glycogen stain, ×1,000: positive Gaucher cells. **(C)** iron stain, ×1,000: positive Gaucher cells (black arrows) and negative Gaucher cells (red arrows).

• Nucleus: Relatively small, with 1–3 or more nuclei that were round or oval and often eccentrically located. The nuclear chromatin appeared coarse.
• Cytoplasm: Abundant, exhibiting a gray-blue or light pink hue, with numerous blue-purple wavy structures resembling onion skin or spider-web patterns (Figure 1A black arrow).
• Special Staining Results:
• Periodic Acid-Schiff (PAS) Staining: Revealed red fibrous filamentous structures within the cytoplasm of Gaucher cells (Figure 1B black arrow).
• Iron Staining: Demonstrated blue fibrous filamentous structures, with some appearing as blue granules, beads, or irregular masses (Figure 1C black arrow). In contrast, iron-free macrophages showed negative staining (Figure 1C red arrow).

### 2.4 Whole spine MRI examination

**Figure 2:**
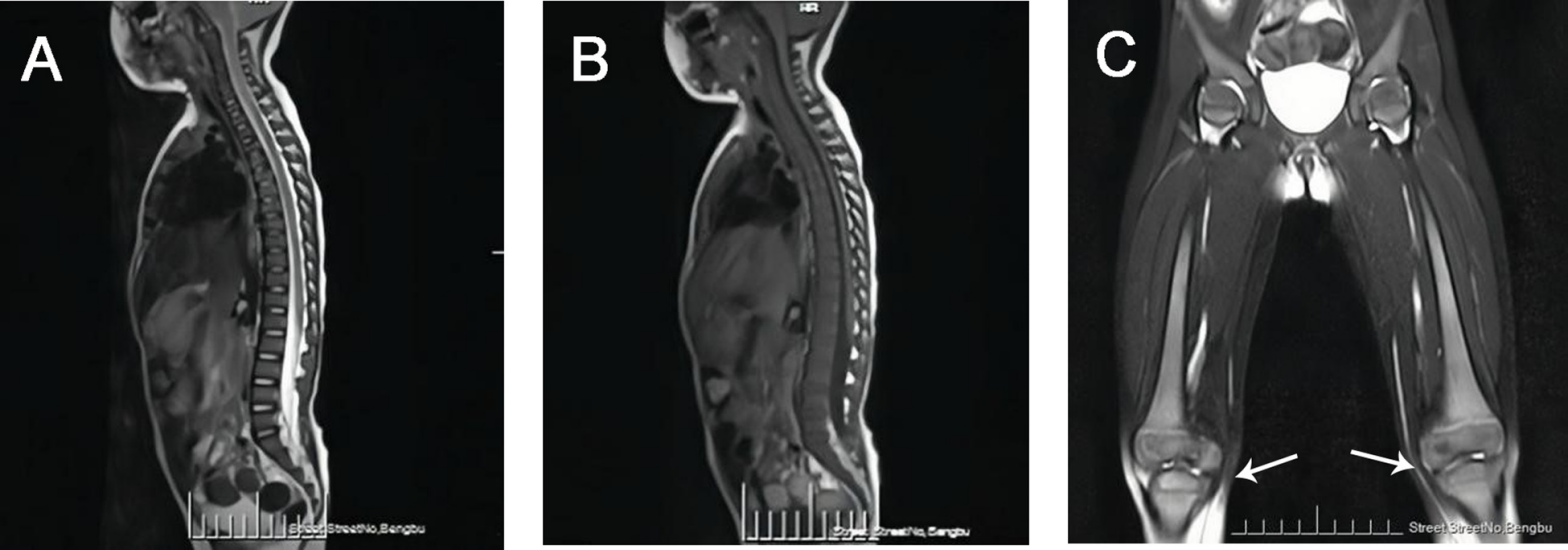
**(A)** MRI-T2WI image demonstrated diffusely decreased signal intensity in the vertebral bodies. **(B)** MRI-T1WI image demonstrated diffusely decreased signal intensity in the vertebral bodies. **(C)** Bilateral femoral MRI demonstrated metaphyseal widening of the distal femora with flask-shaped morphological alterations (white arrow).

The whole spine MRI revealed the following findings:

• Vertebral Alignment and Curvature: The vertebral alignment was normal, and the physiological curvature was preserved.
• Vertebral Signal Intensity: A diffuse reduction in signal intensity was observed throughout the vertebral bodies (Figures 2A,B).
• Femoral Bone Signal Intensity: The signal intensity of the bilateral femoral bones was within normal limits. However, the metaphyseal regions of the distal femurs appeared broadened, exhibiting a flask-like deformity, which is consistent with the radiographic manifestations of Gaucher disease (Figure 2C).
• Hip Joints: A small amount of fluid was noted in both hip joints. No other abnormalities were detected.

### 2.5 Neurological examination

• Video Electroencephalogram (EEG): The results indicated a normal EEG pattern, with no evidence of abnormal electrical activity.
• Auditory Brainstem Response (ABR): The test demonstrated normal responses in both ears, indicating intact auditory pathways and brainstem function.

### 2.6 β-glucocerebrosidase activity, genetic, and biomarker testing

Analysis of the patient's peripheral blood using tandem mass spectrometry revealed significantly decreased β-glucocerebrosidase activity and markedly elevated lyso-Gb1 levels. This specific testing was not performed on either parent. This assay, using pediatric patients and their parents as the test subjects, employed high-throughput sequencing (NGS) to detect single nucleotide variants (SNVs) and small insertions/deletions (indels) (≤30 bp) within the coding regions and adjacent splice regions (approximately ±20 bp) of 445 genes associated with 502 monogenic disorders. The results identified a homozygous missense variant in the patient's *GBA* gene. Detailed results are summarized in Table 2.

**Table 2:** GBA gene variant and enzymatic testing results.

Relationship	GBA gene variant	Zygosity	Variant classification	GBA activity (μmol/L/h)	Lyso-Gb1 level (ng/ml)
				1.19∼22.2	<17.41
Patient	c.1448T>C (p.Leu483Pro)	Homozygous	Pathogenic	0.68	>400
Mother	c.1448T>C (p.Leu483Pro)	Homozygous	Pathogenic	Not tested	Not tested
Father	c.1448T>C (p.Leu483Pro)	Homozygous	Pathogenic	Not tested	Not tested

## 3 Discussion

Gaucher disease (GD) is a rare autosomal recessive disorder classified as a lysosomal storage disorder. It arises from pathogenic mutations in the *GBA* gene encoding β-glucocerebrosidase activity leading to reduced or absent enzymatic activity. This deficiency results in the pathological accumulation of glucocerebroside within the mononuclear-macrophage system, forming characteristic Gaucher cells. Consequently, multiple organs and systems—including the liver, spleen, bone marrow, lymph nodes, skeletal system, and central nervous system—may become progressively involved. According to the presence or absence of nervous system involvement, GD is mainly divided into three types: Type I (Non-neuronopathic GD): This is the most common form, characterized by hepatosplenomegaly, cytopenia, and varying degrees of bone disease, without primary central nervous system involvement. It can occur in all age, with about two-thirds of patients presenting in childhood. A classic skeletal manifestation in children is the “Erlenmeyer flask” deformity, characterized by reduced density and widened metaphyses of long bones. Type II (Acute Neuronopathic GD): In addition to the features of Type I, Type II patients exhibit acute neurological involvement, such as bulbar palsy, oculomotor apraxia, seizures, opisthotonus, and cognitive impairment. This form typically presents in the neonatal to infantile period, progresses rapidly, and is associated with significant developmental delays and a high mortality rate, often leading to death before 2–4 years of age. Type III (Chronic or Subacute Neuronopathic GD): This form shares early features with Type I but gradually develops neurological symptoms, such as oculomotor apraxia, ataxia, opisthotonus, seizures, and myoclonus. It typically presents in childhood, accompanied by developmental delays and intellectual disability. The disease progresses slowly, and patients may have a longer lifespan compared to Type II.

The diagnosis of Gaucher disease (GD) requires comprehensive assessment. Clinical manifestations, laboratory investigations, and imaging studies serve as screening tools, enzymatic activity assays provide diagnostic confirmation, and genetic testing offers definitive verification. Historically, the measurement of glucocerebrosidase enzyme activity in peripheral blood leukocytes has been considered the “gold standard” for diagnosing GD. A diagnosis of GD is confirmed when the enzyme activity falls below 30% of the normal reference range (7, 8). Recent research indicates that measuring Lyso-Gb1 (glucosylsphingosine) in dried blood spots (DBS) is a potential biomarker for GD diagnosis, demonstrating higher sensitivity and specificity (9–11). Ultimately, the identification of pathogenic GBA gene variants through genetic testing provides direct diagnostic confirmation of GD. Other auxiliary diagnostic methods include: Abdominal Ultrasound: Used to evaluate the presence and extent of hepatosplenomegaly. x-ray Imaging: Helpful in assessing osteoporosis, osteonecrosis, or fractures. Magnetic Resonance Imaging (MRI) and bone marrow cytological examination: To detect bone marrow infiltration. Characteristic MRI findings include the “Erlenmeyer flask deformity” of the femur metaphysis. Bone marrow examination may reveal the presence of numerous Gaucher cells. Although bone marrow cytological examination is an invasive procedure, it retains significant clinical utility. Patients often present with unexplained hepatosplenomegaly, anemia, and thrombocytopenia. Clinicians frequently include bone marrow examination as part of the routine workup to investigate cytopenias or rule out leukemia. The unexpected or initial discovery of Gaucher cells in the marrow can point towards a diagnosis of GD, guiding subsequent specific diagnostic tests. Crucially, the presence of Gaucher-like cells in the marrow necessitates differentiation from other conditions: Diseases with similar clinical presentations: Such as Niemann-Pick disease and sea-blue histiocytosis. While their clinical features overlap, Niemann-Pick histiocytes have cytoplasm filled with lipid droplets giving a foamy appearance, whereas sea-blue histiocytes contain cytoplasm packed with large, deep-blue granules. Diseases that may produce Gaucher-like cells in the marrow: For example, chronic myelogenous leukemia (CML) (12).

The genotype of *GBA* gene mutations is correlated with the phenotype. *GBA* gene mutations are highly diverse, including missense mutations (the most frequent type), nonsense mutations, and insertion/deletion mutations (indels), among others. To date, over 500 GBA mutations have been reported both in domestically and internationally. The four most common genotypes are c.1226A>G(p.Asn370Ser), c.84_85insG(p.Val29GlyfsTer?), c.1448T>C(p.Leu483Pro), and c.475C>T(p.Arg120Trp) (12). In Europe and North America, c.1226A>G(p.Asn370Ser) is the most prevalent genotype, accounting for approximately 70% of mutant alleles (13, 14). In contrast, c.1448T>C(p.Leu483Pro) is the most common genotype in China, representing about 33% of mutant alleles (15). Studies have shown that certain mutation types, such as c.84_85insG(p.Val29GlyfsTer?) and c.1448T>C(p.Leu483Pro), are associated with disease severity. For example, patients with type III GD often carry homozygous c.1448T>C(p.Leu483Pro) mutations, while heterozygous c.1448T>C(p.Leu483Pro) mutations are typically associated with type I GD, which lacks neurological involvement (16). Interestingly, the patient in this case harbored a homozygous c.1448T>C(p.Leu483Pro) mutation but exhibited no neurological abnormalities, which appears contradictory to previous findings. However, research by Chinese scholars (13), such as Qu Xianfeng et al, has demonstrated that GD patients with similar clinical manifestations can have different genotypes, and those with the same genotype may present with diverse clinical features. Therefore, based on the clinical presentation, laboratory findings, and genetic testing, a definitive diagnosis of pediatric GD was established for this patient. However, distinguishing between type I and type III GD remains challenging. The patient may have type I GD or could potentially develop neurological abnormalities later, indicating early-stage type III GD. Further genetic testing of the patient's parents revealed that both were heterozygous carriers of the c.1448T>C(p.Leu483Pro) mutation, consistent with Mendelian inheritance.

Current treatment strategies for GD include: Symptomatic Management: Such as blood transfusions, splenectomy, and skeletal interventions to alleviate symptoms. Splenectomy is often used as the main treatment for adult-type GD. Etiological Therapies: Enzyme Replacement Therapy (ERT): The primary drugs available are imiglucerase and velaglucerase alfa, which are used to treat type I and type III GD. Studies have shown that ERT can reverse visceral damage, reduce hepatosplenomegaly, alleviate bone lesions, correct anemia and thrombocytopenia, and optimize growth. However, the need for lifelong intravenous administration remains a limitation (17). Substrate Reduction Therapy (SRT): Oral glucosylceramide synthase inhibitors can significantly improve hepatosplenomegaly but are less effective in addressing thrombocytopenia and bone involvement. Bone Marrow Transplantation: This approach involves implanting healthy hematopoietic stem cells into the patient to increase β-glucocerebrosidase activity. However, challenges such as graft-vs.-host reactions limit its application. The patient continues to be followed up and is currently willing to receive regular intravenous imiglucerase therapy at our hospital.

## 4 Conclusions

GD often presents with an insidious onset and nonspecific clinical manifestations, which overlap with those of various hematological disorders, making it prone to misdiagnosis or missed diagnosis and potentially delaying optimal treatment timing. In clinical practice, when patients exhibit unexplained hepatosplenomegaly, thrombocytopenia, anemia, or other related symptoms that show no significant improvement after a period of symptomatic treatment, GD should be highly suspected. Patients should undergo prompt enzymatic and genetic testing to achieve early detection, diagnosis, and treatment, thereby improving prognosis and extending survival. Concurrently, to mitigate the risk of offspring inheriting this disorder, prospective parents should undergo comprehensive genetic counseling prior to conception. This facilitates assessment of parental carrier status for disease-associated variants, evaluation of offspring recurrence risk, and acquisition of professional reproductive guidance. Implementation of such counseling may alleviate the associated economic burdens at both familial and societal levels.

## Data Availability

The original contributions presented in the study are included in the article/Supplementary Material, further inquiries can be directed to the corresponding author.
